# Polydopamine Nanohydrogel Decorated Adhesive and Responsive Hierarchical Microcarriers for Deafness Protection

**DOI:** 10.1002/advs.202407637

**Published:** 2025-01-17

**Authors:** Hong Chen, Hui Zhang, Jiayi Li, Xianmei Wei, Chenjie Yu, Yuanjin Zhao, Maoli Duan, Xiaoyun Qian, Xia Gao

**Affiliations:** ^1^ Department of Otolaryngology Head and Neck Surgery Nanjing Drum Tower Hospital Jiangsu Provincial Key Medical Discipline Nanjing University Medical School Nanjing 210008 China; ^2^ Research Institute of Otolaryngology No. 321 Zhongshan Road Nanjing 210008 China; ^3^ Department of Rheumatology and Immunology Nanjing Drum Tower Hospital School of Biological Science and Medical Engineering Southeast University Nanjing 210096 China; ^4^ Department of Otolaryngology Head and Neck Surgery & Audiology and Neurotology Karolinska University Hospital Karolinska Institute Stockholm 17176 Sweden; ^5^ Department of Clinical Science Intervention and Technology Karolinska Institute Stockholm 17176 Sweden

**Keywords:** cisplatin, drug delivery, hearing loss, microfluidics, polydopamine

## Abstract

Cisplatin‐induced ototoxicity is attributed to the aberrant accumulation of reactive oxygen species (ROS) within the inner ear. Antioxidants represented by α‐lipoic acid (ALA) have been demonstrated to scavenge ROS in the cochlea, while effective delivery of these agents in vivo remains a major challenge. Here, a novel polydopamine (PDA) nanogel decorated adhesive and responsive hierarchical microcarriers for controllable is presented ALA delivery and deafness prevention. As the composite microcarriers exhibit excellent bioadhesion and responsive hierarchical capabilities, this can result in a prolonged retention time in vivo and allow for precise ALA delivery under light stimulation, which significantly enhances the therapeutic efficacy of ALA. Based on these features, both in vitro and in vivo experiments are demonstrated that the composite microcarriers combined with near‐infrared light irradiation can effectively attenuate cisplatin‐induced ototoxicity. These findings suggest that composite microcarriers are a viable approach for both inner ear medication delivery and the prevention of deafness.

## Graphical abstract

A type of polydopamine (PDA) nanogel decorated adhesive and responsive hierarchical microcarriers for ALA delivery and deafness prevention. PDA@microcarriers can protect the encapsulated drugs from external disturbances and prolong their retention time in the inner ear. Besides, the responsive hierarchical properties of PDA@microcarriers enabled them to deliver the drug to the inner ear in a controlled manner under light triggering.

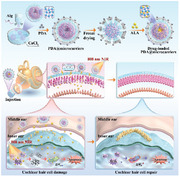

## 1 Introduction

Deafness triggered by various factors such as infections, hypoxia, noise exposure, and ototoxic drug abuse (cisplatin, gentamicin, neomycin, etc.) is becoming increasingly prevalent worldwide.^[^ ^1^ ^]^ Particularly, cisplatin‐induced ototoxicity causes hair cell apoptosis and the permanent death of auditory cells, resulting in bilateral, gradual, and irreversible sensorineural hearing loss.^[^ ^2^ ^]^ Antioxidants have been shown in several studies to be able to shield cochlear hair cells against ototoxicity caused by cisplatin.^[^ ^3^ ^]^ Among various existing antioxidants, α‐lipoic acid (ALA), a cofactor of mitochondrial α‐keto acid oxidative dehydrogenase, can ameliorate cochlear injury through various mechanisms.^[^ ^4^ ^]^ For instance, ALA can inhibit the activation of the programmed cell death pathway and necroptosis by scavenging oxygen radicals, chelating metal ions and mobilizing the production of endogenous antioxidants.^[^ ^5^ ^]^ However, traditional systemic administration of ALA faces multiple obstacles, especially the blood labyrinth and membrane labyrinth barriers that impede the entry of ALA into the inner ear, resulting in suboptimal therapeutic efficacy.^[^ ^2^, ^6^ ^]^ Thus, developing an inner ear ALA delivery system with precise targeting and sustained drug delivery is still anticipated.

**Figure 1:**
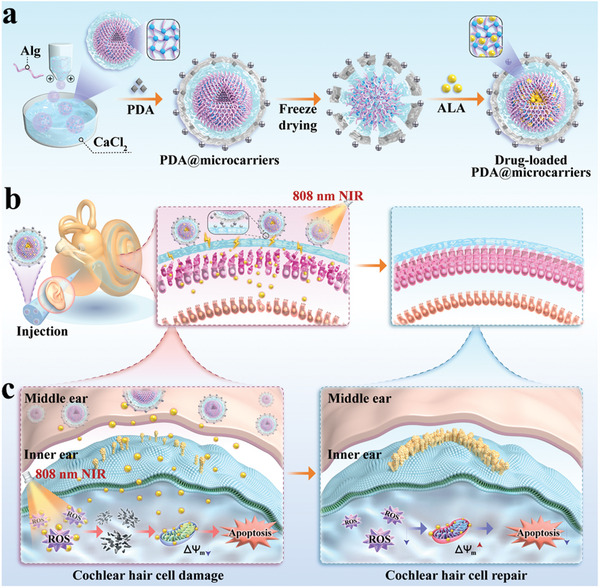
Schematic diagram of PDA nanohydrogel decorated adhesive and responsive hierarchical microcarriers for ALA delivery and deafness prevention. a) Fabricating PDA@microcarriers loaded with ALA. Alg refers to the sodium alginate. b) Injecting PDA‐modified microcarriers (PDA@microcarriers) into the middle ear and irradiating by near‐infrared ray (NIR) light. c) The mechanism by which loaded ALA enters the inner ear to exert its effects.

Here, a novel bionic adhesive and responsive hierarchical alginate microcarriers decorated with polydopamine (PDA) nanogel and loaded with ALA are designed for controllable ALA delivery and deafness prevention, as schemed in **Figure** **1**. In nature, multifarious organisms such as mussels exhibit excellent adhesion properties based on molecular attraction. Inspired by this feature of mussels, PDA has attracted extensive attention due to its structural similarity to mussel‐secreted adhesive protein.^[^ ^7^ ^]^ PDA‐incorporated materials are thus capable of tightly attaching to various substrates through noncovalent and covalent interactions. Additionally, PDA displays attractive photothermal conversion capability, further expanding the scope of its applications in the biomedical field.^[^ ^8^ ^]^ In contrast, microfluidics has been widely employed for the continuous fabrication of microcarriers with adjustable size and functionality for drug encapsulation and delivery based on its ability to precisely control and manipulate tiny fluids.^[^ ^9^ ^]^ Therefore, we envisioned that combining adhesive PDA with microfluidic microcarriers would lead to the development of new systems with the desired properties for the local delivery of ALA.

In this paper, we fabricated the desired ALA‐loaded microcarriers with large specific surface areas and porous structures using droplet microfluidics and PDA nanogel modification. The rough surface of these microcarriers facilitated cell adhesion while the porous structures allowed them for slow release of the loaded ALA and substance exchange. Benefitting from PDA modification, the obtained microcarriers were endowed with remarkable bioadhesive and responsive hierarchical properties. These bioadhesive PDA@microcarriers could protect the encapsulated drugs from external disturbances and prolong their retention time in the inner ear. Besides, the responsive hierarchical properties of PDA@microcarriers enabled them to deliver the drug to the inner ear in a controlled manner under light triggering, thus prolonging the drug action time. In vitro studies revealed that ALA‐loaded composite microcarriers could effectively protect HEI‐OC1 cells from cisplatin‐induced oxidative stress damage by reducing the accumulation of reactive oxygen species (ROS) and decreasing the mitochondrial membrane potential (MMP). In an animal model of cisplatin‐induced deafness, the proposed composite microcarriers successfully accelerated ALA entry into the inner ear upon photo triggering, thereby reducing the incidence of cisplatin‐induced ototoxicity. These results bring hope for the auditory preservation of cisplatin‐induced ototoxicity patients in the clinic.

## 2 Results and Discussion

### 2.1 Preparation and Characterization of PDA@microcarriers

**Figure 2:**
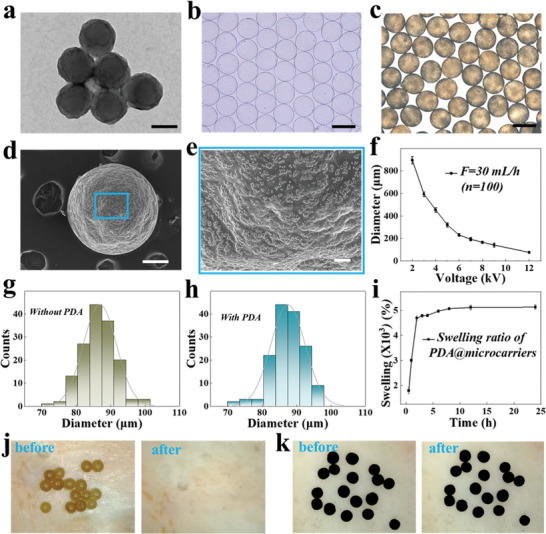
Characterization of the PDA@microcarriers. a) Transmission electron microscopy image of PDA nanoparticles. Scale bar: 100 nm. b,c) Optical image of microcarriers before (b) and after (c) loading with PDA nanoparticles. Scale bars: 100 µm. d,e) SEM pictures of PDA@microcarriers from global (d) and local perspective (e). Scale bars: 20 µm. f) Statistical analysis of the diameter of PDA@microcarriers at various voltages. The flow rate is 30 mL h^−1^. g,h) Statistical particle size distribution of microcarriers without (g) and with PDA (h) at the flow rate of 30 mL h^−1^ and the voltage of 12 kV. i) Swelling curves of PDA@microcarriers in PBS (n = 3). j,k) Pictures of microcarriers j) and PDA@microcarriers k) retained on the skin surface before and after shaking in PBS.

The adhesive and photo‐responsive microcarriers were prepared through microfluidics and PDA modification. In detail, dopamine self‐polymerization was used to create PDA nanoparticles, showing an average diameter of 127 ± 7.44 nm and homogeneity (**Figure** **2**a). To obtain microcarriers in high throughput, the microfluidic electrospray technique was utilized, which forms an electric field by applying a high voltage between the capillary glass tube and the receiving liquid. The sodium alginate liquid shot out of the Taylor cone's tip and into the calcium chloride receiving liquid as a result of the intense voltage breaking through the liquid's surface tension and viscosity. When the negatively charged sodium alginate liquid came into contact with the positively charged calcium chloride liquid, a stable 3D spherical structure was formed of microcarriers through electrostatic interactions, as observed in Figure 2b. Of note, the monodispersity and yield of these microcarriers were significantly more efficient compared to conventional diffusion control methods. In order to endow the microcarriers with good adhesion properties and photothermal responsiveness capabilities, we modified these microcarriers with PDA nanoparticles through in situ polymerization (Figure 2c). It was observed by scanning electron microscopy (SEM) that a large number of PDA nanoparticles were attached to the surface of microcarriers (Figure 2d,e). Additionally, the PDA@microcarriers of various sizes were generated by regulating the voltage parameters of the microfluidic system (Figure 2f, Figure S1, Supporting Information). It should be noted that the small size is favorable for the success of the procedure when injected into the middle ear using a syringe. Accordingly, the microcarriers with a diameter of 86.9 ± 4.8 µm were selected for the subsequent experiment, while the diameter of PDA@microcarriers was 87.6 ± 5.0 µm (Figure 2g,h). Furthermore, the lyophilized PDA@microcarriers exhibited excellent water adsorption capability, which reached the swelling equilibrium after soaking in phosphate buffer saline (PBS) for 4 h with a swelling degree of ≈4799 ± 54.45%, contributing to the rapid loading of drugs. In particular, the adhesion properties of microcarriers contribute to their long‐term in situ retention within the middle ear cavity. To assess this property, we injected appropriate amounts of microcarriers and PDA@microcarriers into the mouse skin surface, respectively, left it for 10 min, and then rinsed the surface with PBS. As shown in Figure 2j and Movie S1 (Supporting Information), the microcarriers lacking PDA modification exhibited a tendency to dislodge with the flow of PBS, whereas the PDA@microcarriers demonstrated a remarkable ability to remain firmly fixed on the surface even after rinsing and shaking (Figure 2k and Movie S2, Supporting Information). These findings suggest that PDA@microcarriers possess satisfactory adhesion properties.

### 2.2 Photothermal Responsive Property of PDA@microcarriers

**Figure 3:**
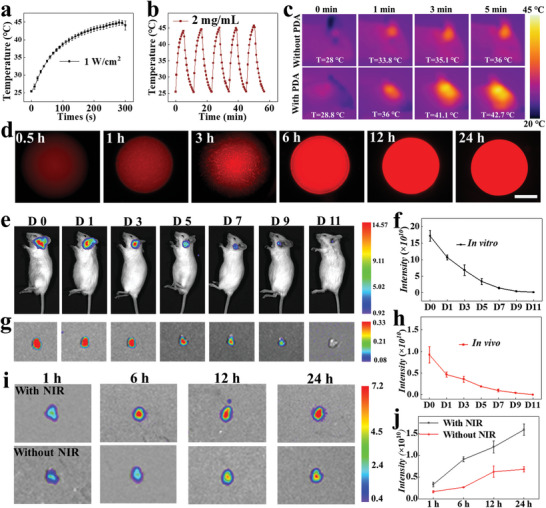
Drug loading and release study. a) Temperature rising curves of PDA@microcarriers modified with 2 mg mL^−1^ PDA nanoparticles under 1W cm^−^^2^ NIR radiation (n = 5). b) Temperature profile of PDA@microcarriers during 5 on/off cycles under 1 W cm^‐^^2^ NIR radiation. c) In situ thermal presentation after injection of microcarriers and PDA@microcarriers in the middle ear of mice. d) Confocal scanning laser microscopy images of PDA@microcarriers loaded with Rhodamine B (RHB) at different time periods. Scale bar: 50 µm. e) In vivo imaging system (IVIS) images of mice treated with cyanine (Cy7)‐loaded PDA@microcarriers after different periods. f) Statistical analysis of the fluorescent intensity of mice (n = 5). g) IVIS images of the isolated cochlea from mice treated with Cy7‐loaded PDA@microcarriers after different periods. h) Statistical analysis of the fluorescent intensity of cochlea (n = 5). i) IVIS images of the cochlea from mice injected with Cy7‐loaded PDA@microcarriers within 24 h (n = 3).

Benefitting from the PDA nanoparticles' capacity to convert photothermally, the generated PDA@microcarriers showed adjustable temperature under NIR irradiation. To find suitable thermo‐responsive microcarriers, we subjected customized microcarriers to varying concentrations of PDA nanoparticles. All concentrations of PDA@microcarriers' temperature increased after 5 min of NIR irradiation (Figures S2 and S3, Supporting Information). Following a 5 min NIR irradiation, it was found that the temperature of PDA@microcarriers modified with PDA nanoparticles at a concentration of 2 mg mL^−1^ increased to ≈40 °C, whereas the temperature of PDA@microcarriers at PDA concentrations higher than 2 mg mL^−1^ increased rapidly over 40 °C (**Figure** **3**a). Considering these findings and the potential risk of thermal damage to neighboring tissues at high temperatures, 2 mg mL^−1^ and 1 W cm^−^^2^ were determined to be the appropriate PDA nanoparticle concentration and NIR energy, respectively. To evaluate the controllability of NIR light on the aforementioned photothermal properties of PDA@microcarriers, we exposed PDA@microcarriers to 5 on/off cycles of NIR light irradiation. In each cycle, the PDA@microcarriers exhibited rapid warming to 40 °C when the NIR light was turned on and cooled back to the initial temperature immediately after the NIR light was turned off (Figure 3b). These results revealed that the local temperature of PDA@microcarriers could be manipulated by adjusting the NIR irradiation. Then, the middle ears of the mice were injected with PDA@microcarriers, which were then subjected to NIR light. It was observed that the local temperature of the PDA@microcarriers‐injected site increased to 42 °C within 5 min after receiving NIR, while that in the control group (intra‐auricular injection of microcarriers without PDA modification) only increased to 36 °C, suggesting that the PDA@microcarriers kept favorable photothermal responsive properties in vivo (Figure 3c).

### 2.3 Drug Loading and Release Behavior of PDA@microcarriers

To determine the drug‐loading behavior of the PDA@microcarriers, we chose RHB instead of ALA as the model drug, and the PDA@microcarriers were observed using confocal microscopy to determine the distribution of the drug. It was noticed from the laser confocal images that RHB exhibiting red fluorescence was evenly distributed throughout the PDA@microcarriers (Figure 3d). Moreover, the fluorescence intensity of the PDA@microcarriers after 24 h loading was comparable to that observed after 12 h loading, suggesting that the drug was completely loaded after 12 h of immersion. To observe the distribution of PDA@microcarriers in the cochlea after administration via the middle ear cavity, Cy7 dye with high tissue penetration and minimal background interference was selected to replace ALA for in vivo tracer observation. The fluorescence signal of PDA@microcarriers loaded with Cy7 was seen to persist for almost 11 days, with a slow decline (Figure 3e,f). Besides, the presence of a fluorescent signal in the isolated cochlea of mice injected with Cy7‐loaded PDA@microcarriers was observed for over 9 days, demonstrating that the drug might continue to enter the inner ear (Figure 3g,h). In contrast, the fluorescence signals in the mice that were directly injected with Cy7 lasted only ≈4 days, and there was almost no fluorescence in their isolated cochlea after 4 days (Figure S4, Supporting Information). These results suggested that PDA@microcarriers increased drug retention time in the middle ear as well as facilitated drug entry into the inner ear, thereby improving drug bioavailability. Furthermore, we examined the controllable drug release property of PDA@microcarriers under NIR irradiation. It was found that the fluorescence intensity of the cochlea after NIR light irradiation was higher than that of the cochlea without NIR irradiation at different time periods (Figure 3i,j). This finding demonstrates that NIR‐responsive characteristics of PDA@mcirocarriers contribute to improved drug entry into the inner ear.

### 2.4 Biocompatibility of PDA@microcarriers

The development of PDA@microcarriers as injectable drug carriers to antagonize cisplatin ototoxicity requires consideration of their biocompatibility, as well as the potential impact of NIR irradiation on cellular activity. Therefore, we used a live‐dead kit to investigate the cytotoxicity of PDA@microcarriers, NIR, and NIR+ PDA@microcarriers on the cochlear hair cell line (HEI‐OC1 cells). On days 1, 2, and 3, HEI‐OC1 cells co‐cultured with PDA@microcarriers, NIR, and NIR+ PDA@microcarriers were morphologically similar to the normal medium without PDA@microcarriers (control group) and grew well (Figure S5, Supporting Information). Obviously, cell proliferation rates in the PDA@microcarriers and NIR groups were consistent with those in the control group, with almost no dead cells in each group. Taken together, the results indicate that the prepared PDA@microcarriers with NIR irradiation have satisfactory biocompatibility, which provides fundamental data support for subsequent in vivo applications.

### 2.5 Scavenging Ability of ALA‐Loaded PDA@microcarriers (PDA@microcarriers‐ALA) to Cisplatin‐Induced ROS

**Figure 4:**
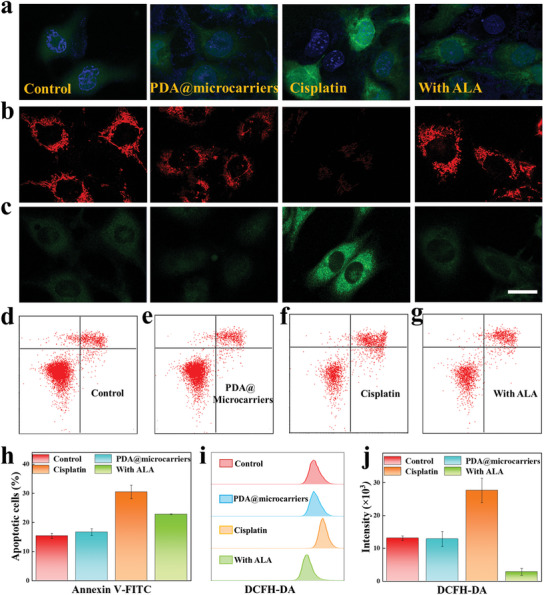
Protective effects of PDA@microcarriers‐ALA on HEI‐OC1 cells. a) DCFH‐DA staining photographs of cells with different treatments. Green: DCFH‐DA; Blue: Hoechst 33258. b,c) JC‐1 images of cells under various conditions. JC‐1 monomer is marked in green, whereas JC‐1 aggregate is marked in red. Scale bar: 20 µm. d–g) HEI‐OC1 cells' apoptosis percentage was examined through an apoptosis detection kit. h) Statistical evaluation of several groups' apoptotic cell populations. i) Flow cytometry analysis of ROS in different groups. j) Mean fluorescence intensity statistics for the ROS in different groups (n = 3).

Following cisplatin exposure, blood flow in the cochlea slows down, resulting in ischemia and hypoxia due to insufficient blood supply to the inner ear. As a result, a large number of free radicals are generated, which directly damage cellular and nuclear DNA through oxidative stress, causing pathological changes such as intracellular calcium ion overload and ultimately leading to cell apoptosis. It has been confirmed that the damage to the inner ear caused by cisplatin‐induced oxidative stress can be effectively mitigated by antioxidants. To assess the antagonizing effect of PDA@microcarriers‐ALA (with ALA group) in oxidative stress injury at the cellular level, we constructed cisplatin‐injured HEI‐OC1 cells and determined ROS levels using 2′,7′‐dichlorofluorescein diacetate (DCFH‐DA). It was found that the cells' fluorescence signal in the cisplatin‐treated group was stronger than the PDA@microcarriers‐ALA pretreatment group. Conversely, the fluorescence signal of the latter group was similar to that of the control and PDA@microcarrier groups (**Figure** **4**a). All the above four groups were irradiated by NIR. We further confirmed this finding by flow cytometry, as shown in Figure 4i,j. These results suggest that PDA@microcarriers‐ALA can effectively scavenge cisplatin‐induced ROS generation.

Mitochondria are one of the major sources of ROS, so damage to the mitochondria may lead to more ROS production, which may damage other parts of the cell, such as lipids, proteins, and DNA. Sustained damage to mitochondria may trigger apoptosis or necrosis, which is a process of cellular self‐destruction. Mitochondrial functions of HEI‐OC1 cells with different treatments were obtained by measuring MMP through the JC‐1 probe. An early sign of apoptosis is a decrease in MMP, which is manifested by the emission of green fluorescence (JC‐1 monomer). It was observed that there was a negligible difference in red fluorescence between the PDA@microcarriers group and the control group, as seen in Figure 4b,c. In contrast, stronger green fluorescence intensity was observed in the cisplatin‐treated group over the other three groups. These findings demonstrate the effective protection of PDA@microcarriers‐ALA on the mitochondrial function of HEI‐OC1 cells.

### 2.6 Antagonistic Impact of ALA‐loaded PDA@microcarriers

Furthermore, in order to assess the pre‐protective impact of PDA@microcarriers‐ALA on cisplatin‐injured cells, we detected apoptosis in each group using the Annexin V‐FITC/PI detection kit. Annexin V binds specifically and with high affinity to phosphatidylserine, which is flipped to the outside of the membrane during apoptosis, whereas PI stains the nuclei of advanced apoptotic and necrotic cells red. Consequently, apoptotic cells may be identified when Annexin V and PI are combined. Following a 24 h co‐cultivation period, the PDA@microcarriers and control groups' apoptosis rates were 16.66 ± 1.15% and 15.39 ± 0.89%, with no statistical difference (Figure 4d,e,h). The apoptosis rate in the cisplatin group was 30.49 ± 2.35%, whereas the rate of apoptosis in the PDA@microcarriers‐ALA pre‐protected group was 22.78 ± 0.14% (Figure 4f,g,h). These results suggest that PDA@microcarriers‐ALA antagonizes the ototoxicity of cisplatin.

### 2.7 Protective Effect Evaluation of PDA@microcarriers‐ALA on Outer Hair Cells (OHCs) Located on the Cochlear Basilar Membrane

**Figure 5:**
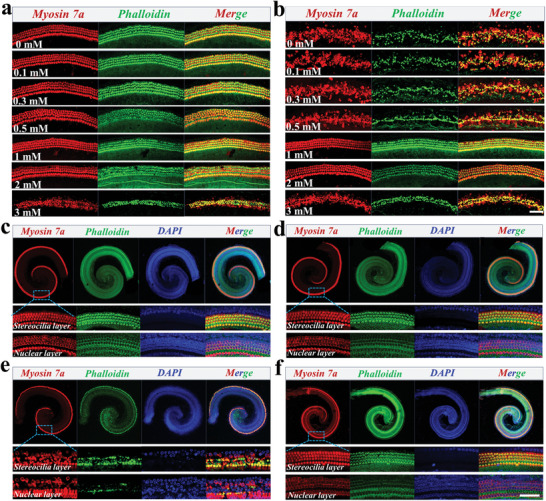
Protective effect evaluation of PDA@microcarriers‐ALA on the cochlear basilar membrane. a) Immunofluorescent staining images of basilar membranes treated with various doses of ALA. b) Immunofluorescent staining images of basilar membranes that were treated with 30 µm cisplatin after being pretreated with various doses of ALA. c–f) Immunofluorescent staining images of basilar membranes in the control group (c), PDA@microcarriers group (d), cisplatin group (e), as well as PDA@microcarriers‐ALA and cisplatin group (f). Scale bars: 50 µm.

Prior to co‐incubation with microcarriers, the cochlear basilar membrane of Postnatal Day 3 (P3) mice was collected and treated with various doses of ALA (**Figure** **5**a). It was found that when the ALA concentration exceeded 1 mm, the OHCs on the basilar membrane were damaged. To further investigate a suitable protective concentration of ALA antagonizing the damaging effects of cisplatin, basilar membranes were pretreated with different doses of ALA for 24 h before being given 30 µm cisplatin for 24 h. As exhibited in Figure 5b,l and 2 mm of ALA had a better protective effect on cells subjected to cisplatin‐induced injury. Based on the aforementioned results, the final concentration of ALA was set at 1 mm for the subsequent experiments. Furthermore, isolated basilar membranes were divided into four groups, which were treated with normal culture medium (control group), PDA@microcarriers, 30 µm cisplatin, PDA@microcarriers‐ALA and 30 µM cisplatin, respectively. Consequently, pure PDA@microcarriers did not damage the OHCs on the basilar membrane compared with the control group, indicating good biocompatibility of PDA@microcarriers (Figure 5c,d). Besides, 30 µm cisplatin caused heavier damage to the OHCs on the basilar membrane, whereas PDA@microcarriers‐ALA demonstrated a notable protective effect on the OHCs on the basilar membranes from cisplatin‐induced injury (Figure 5e,f).

### 2.8 Hearing Protection Against Cisplatin Ototoxicity by PDA@microcarriers‐ALA

**Figure 6:**
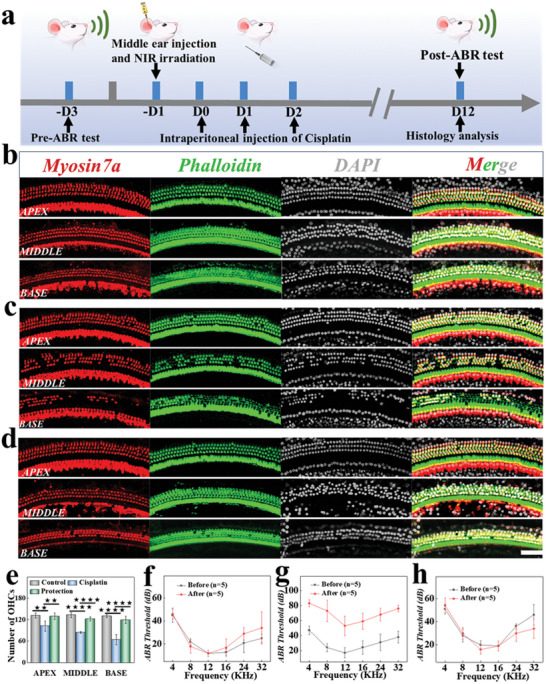
Hearing protection against cisplatin ototoxicity. a) Schedule for animal experiments. b–d) Immunofluorescence pictures of the control (b), cisplatin (c), and protection (d) groups' apical, middle, and basal segments. e) Statistics of surviving OHCs in each group. f–h) ABR thresholds of mice in the control (f), cisplatin (g), and Protection (h) groups before and after the intervention (n = 5). Scale bar: 50 µm. (^★★^ *p* <0.01; ^★★★★^ *p* < 0.0001).

The prepared PDA@microcarriers‐ALA exhibited compact size and injectability, rendering them ideal candidates for middle ear administration. For in vivo evaluation, adult mice were randomly assigned to control, cisplatin, and the PDA@microcarriers‐ALA (Protection) groups, with five mice in each group. The PDA@microcarriers‐ALA group received an injection of PDA@microcarriers‐ALA through the tympanic membranes. A dose of ≈10 uL was injected into each ear. Subsequently, cisplatin was intraperitoneally injected into the mice in the cisplatin group and Protection group for a period of 3 days, as illustrated in **Figure** **6**a. 10 days post‐surgery, basilar membranes of mice in all groups were collected and stained with myosin VIIa, phalloidin and DAPI. Mice's OHCs were shown to be severely damaged by cisplatin therapy; however, PDA@microcarriers‐ALA injection was found to be successful in shielding OHCs from cisplatin ototoxicity, with results similar to those of the control group (Figure 6b–e).

The electrophysiologic indicator known as the auditory brainstem response (ABR) is frequently employed in hearing assessments and has been utilized extensively in fundamental auditory research. Thus, in order to evaluate the hearing capacity of the experimental mice, ABR thresholds were recorded 3 days prior to treatment and 10 days following injection. The results revealed that there was no change in hearing in the control group, indicating that tympanic membrane perforation modeling did not impair hearing in mice (Figure 6f). In contrast, the cisplatin group showed significant hearing loss with higher ABR thresholds after 10 days of cisplatin treatment (Figure 6g), but the mice in the Protection group' ABR thresholds had marginally altered (Figure 6h). These findings imply that PDA@microcarriers effectively transport loaded ALA to the inner ear, reducing cisplatin‐induced hearing loss.

## 3 Conclusion

In summary, we propose novel PDA nanohydrogel decorated adhesive and responsive hierarchical microcarriers for the controllable delivery of ALA and the prevention of deafness. The manufactured microcarriers' porous nature and sizable surface area enable the loading and release of the medication. Furthermore, the adding of PDA endows the composite microcarriers with remarkable bioadhesion and responsiveness to NIR irradiation, thus prolonging the retention time of microcarriers in vivo and enabling the precise delivery of ALA into the ear, which significantly improves the therapeutic efficacy of ALA. In vitro experiments confirm their excellent biocompatibility and satisfactory antioxidant ability against HEI‐OC1 cells and basement membrane cells. According to the findings of in vivo experiments, the prepared ALA‐loaded PDA@microcarriers combined with NIR can effectively attenuate cisplatin‐induced ototoxicity and reduce the loss of OHCs. These findings imply that composite microcarriers represent a promising approach for preventing deafness and delivering drugs into the inner ear.

## 4 Experimental Section

### Preparation of PDA@microcarriers

First, 2 wt.% high‐viscosity sodium alginate was dissolved and inhaled into a syringe. Then, various voltages were applied using a voltage generator, which could influence the size of the generated droplets. Under the action of an electric field, the ejected liquid was transformed into Taylor's cone and subsequently collected in a vessel containing a 2 wt.% calcium chloride solution. The microcarriers were then placed in a shaker and immersed in a solution of Tris‐HCl (10 mM, pH 8.5) that contained different amounts of dopamine. After 24 h of shaking at 37 °C in the dark, PDA@microcarriers were obtained. The sphericity of the microcarriers was observed, the particle size distribution was counted using an optical microscope, and the microstructure was characterized using a field emission scanning electron microscope.

### Swelling Test

First, a 5 mL centrifuge tube was filled with 0.01 g of lyophilized PDA@microcarriers, which had been previously weighed. After that, centrifuge tube was put on a shaker set to 200 rpm min^−1^ at 37 °C, and 1 mL of PBS was added. The supernatant was then removed and the residuum was weighed. At various time intervals, the supernatant was finally extracted, and the PDA@microcarriers were gathered for further weighing. The swelling ratio was calculated as follows:

(1) $$\mathrm{Swelling}\;\mathrm{ratio}\;=\left(\mathrm{wet}\;\mathrm{weight}\;-\;\mathrm{dry}\;\mathrm{weight}\right)\;\;/\;\mathrm{dry}\;\mathrm{weight}\;\times 100\%$$

### Drug Loading

First, to simulate the loading rate of small molecule drugs, RHB was employed at a dissolving concentration of 1 mg mL^−1^. Then, RHB solution was dipped into the PDA@microcarriers that had been manufactured. Subsequently, the PDA@microcarriers were collected at various intervals and observed by confocal microscopy.

### Drug Release

The prepared PDA@microcarriers were infiltrated into a well‐dissolved 1 mg mL^−1^ Cy7 solution, and the mice in the experimental group were injected with a specific volume of PDA@microcarriers‐Cy7 solution. In contrast, a specific amount of Cy7 solution was injected into the mice in the control group. Subsequently, mice and isolated cochleae from varying time points were obtained and observed by IVIS.

### Biocompatibility Assay of PDA@microcarriers

HEI‐OC1 cells were used to test the cytotoxicity of PDA@microcarriers. The cells in the control group were treated with a normal culture medium, while the cells in the PDA@microcarriers group were co‐cultured with PDA@microcarriers. Following a 30‐min treatment with a live/dead staining kit diluted with PBS  at a ratio of 1:1000 on days 1, 2, and 3, confocal images were captured.

### Mitochondrial Function Assay

In 6‐well plates, HEI‐OC1 cells were seeded and split into four groups. The cells in the control group received no treatment, while the cells in the cisplatin group and PDA@microcarriers group were treated with 30 µm cisplatin and PDA@microcarriers, respectively. Besides, in the PDA@microcarriers‐ALA group, the cells were pretreated with PDA@microcarriers containing ALA for 12 h, followed by a 24 h treatment with 30 µm cisplatin. All the above four groups were irradiated by NIR. Then, the medium from each group was removed, and the cells were washed by adding 1 mL of PBS to each well. PBS and DCFH‐DA were mixed 1000:1, added to different groups of cell culture plates, and incubated for 20 min. After that, Hoechst 33258 was introduced to the plates for incubation. Ultimately, the confocal microscope was used to examine each set of cells. Furthermore, MMP Assay Kit was used to evaluate the mitochondria's function in different cell groups. Different subgroups of cells were stained and visualized under a Zeiss laser confocal microscope according to the instructions of MMP.

### Flow Cytometry

The cells were divided into four groups in accordance with the above grouping. After trypsin digestion, the cells were collected into centrifuge tubes. The supernatant was extracted after the cells in the centrifuge tube were spun for 5 min at 1000 rpm. After thoroughly mixing Annexin V‐FITC into each set of cells, PI was added, and the cells were incubated. The results of different groups of cells in the lower and upper right quadrants were detected by flow cytometry and counted by GraphPad software.

### Establishment of Animal model

Mice aged 7–8 weeks and weighing 25–30 g were selected for the study. The external ear canal and middle ear of the experimental mice were examined to ensure that the hearing of the experimental mice was normal. Any mice with abnormal external ear canal and otitis media were excluded. Three groups of mice were assigned at random. The cisplatin group received an intraperitoneal injection of cisplatin, while the control group underwent a tympanic membrane puncture. In the PDA@microcarriers‐ALA group, cisplatin was injected intraperitoneally one day after the injection of PDA@microcarriers‐ALA into the ear. ABR assay and histological analysis were performed on mice to assess the pre‐protective function of PDA@microcarriers‐ALA. The Nanjing Drum Tower Hospital's Animal Ethics Committee has approved all mouse‐based animal research (2023AE01003).

### ABR Testing

The mice were anesthetized with 10 mg mL^−1^ sodium pentobarbital. Next, the audiometric machine was turned on and set up in a soundproof room. Then, the electrodes were inserted subcutaneously into the mice in the correct order. The audiometric software was turned on and the frequencies tested were 4–32 kHz. 90 dB was used as the starting point for the test, and the thresholds were recorded by decreasing the frequency by 5 dB each time from low to high. Finally, the results were analyzed using GraphPad software.

### Immunofluorescence Staining

Cochleae from P3 mice were collected into a four‐well dish and fixed by 4 wt.% paraformaldehyde (PFA) for 1 h. Following three 20 min washes with 1 vol% PBST (PBS containing 1 vol% Triton X‐100), the cochleae were blocked for 1 h using 90 µL of blocking media. Then, proportionally diluted rabbit‐derived anti‐myosin VIIa (Myosin7a) was added into each well and incubated. After washing three times, the diluted amounts of DAPI, Phalloidin‐488 (ph488), and Goat anti‐rabbit IgG Alexa Fluor 555 were added the next day, and the mixture was added to the well to incubate for 2 h at room temperature and away from light. Finally, the cochleae were washed three times, sealed by adding an anti‐fluorescence quencher and stored at 4 °C in a refrigerator protected from light.

In adult mice, the basilar membrane of the cochlea was dissected out of the temporal bone under a microscope, and the top of the cochlea was punched and fixed in 4 wt.% PFA for 1 day. Ethylene diamine tetraacetic acid (0.5 m) was used to decalcify the fixated temporal bone for 3 days. The cochlear basilar membrane was then peeled off under a microscope and the cochlea was collected in a container with a circular coverslip. The remaining steps were consistent with the P3 basilar membrane.

### Statistical Analysis

All statistical data were expressed as mean ± standard deviation. GraphPad was employed to analyze all data, while Origin was utilized to plot. ANOVA was used to evaluate data involving three or more groups. Flowjo software was used to process the flow analysis findings. At *p* < 0.05, statistical significance was established.

## Conflict of Interest

The authors declare no conflict of interest.

## Author Contributions

H.C., H.Z. and J.Y.L. contribute equally. The experiment was planned and the idea originated with C.J.Y., X.G., X.Q., M.L.D., and Y.J.Z. H.C. carried out data analysis and experimentation. The manuscript was written by H.C. and H.Z. J.Y.L. and X.M.W. helped write the paper.

## Supporting information



Supporting Information

Supplemental Movie 1

Supplemental Movie 2

## Data Availability

The data that support the findings of this study are available from the corresponding author upon reasonable request.
