# Supplementary material for: Polydopamine Nanohydrogel Decorated Adhesive and Responsive Hierarchical Microcarriers for Deafness Protection
Source: Adv Sci (Weinh). 2025 Jan 17;12(29):2407637. doi: 10.1002/advs.202407637 (PMC12362749; doi:10.1002/advs.202407637)
Supplement: Supplementary file 1 — Supporting Information [file ADVS-12-2407637-s002.docx]

Supporting Information

Polydopamine Nanohydrogel Decorated Adhesive and Responsive Hierarchical Microcarriers for Deafness Protection

Hong Chen, Hui Zhang, Jiayi Li, Xianmei Wei, Chenjie Yu*, Yuanjin Zhao*, Maoli Duan*, Xiaoyun Qian*, Xia Gao*


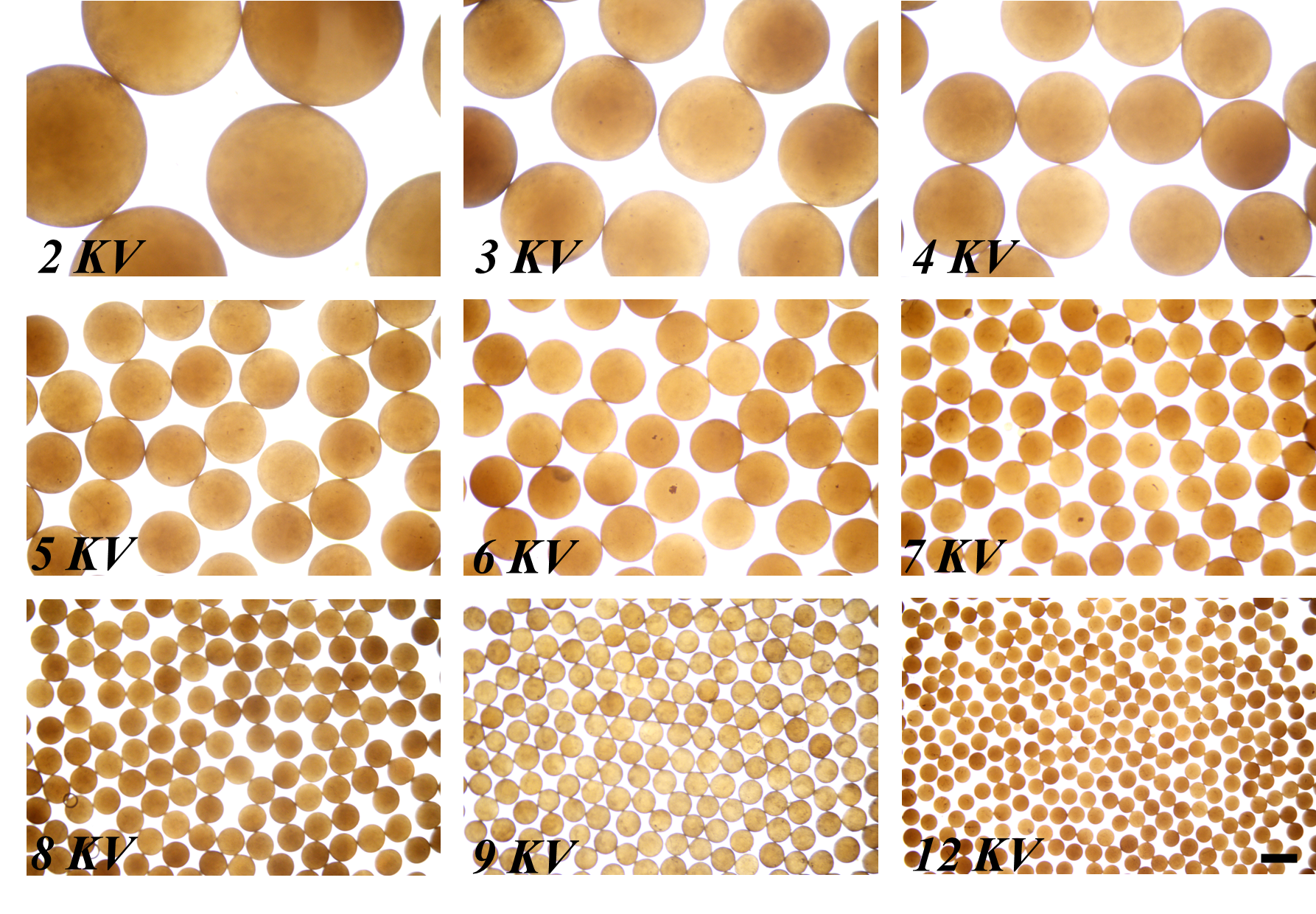


**Figure S1** Optical images of PDA@microcarriers at different voltages. Scale bar: 200 μm.

**Figure S2** Photothermal results of PDA@microcarriers with different PDA concentrations under 1W/cm^2^ NIR light.


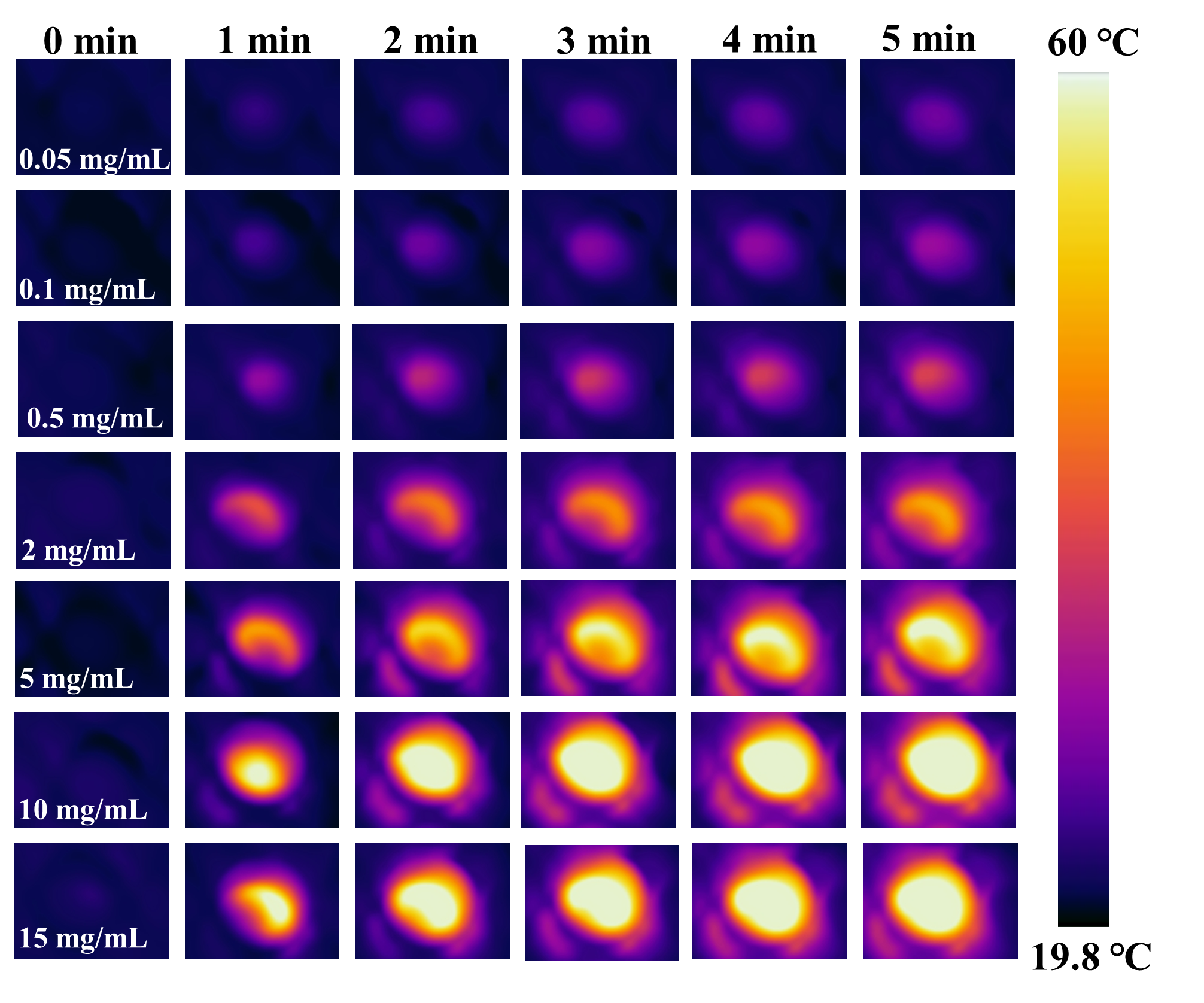


**Figure S3** Photothermal pictures of PDA@microcarriers with different PDA concentrations under 1W/cm^2^ NIR light.


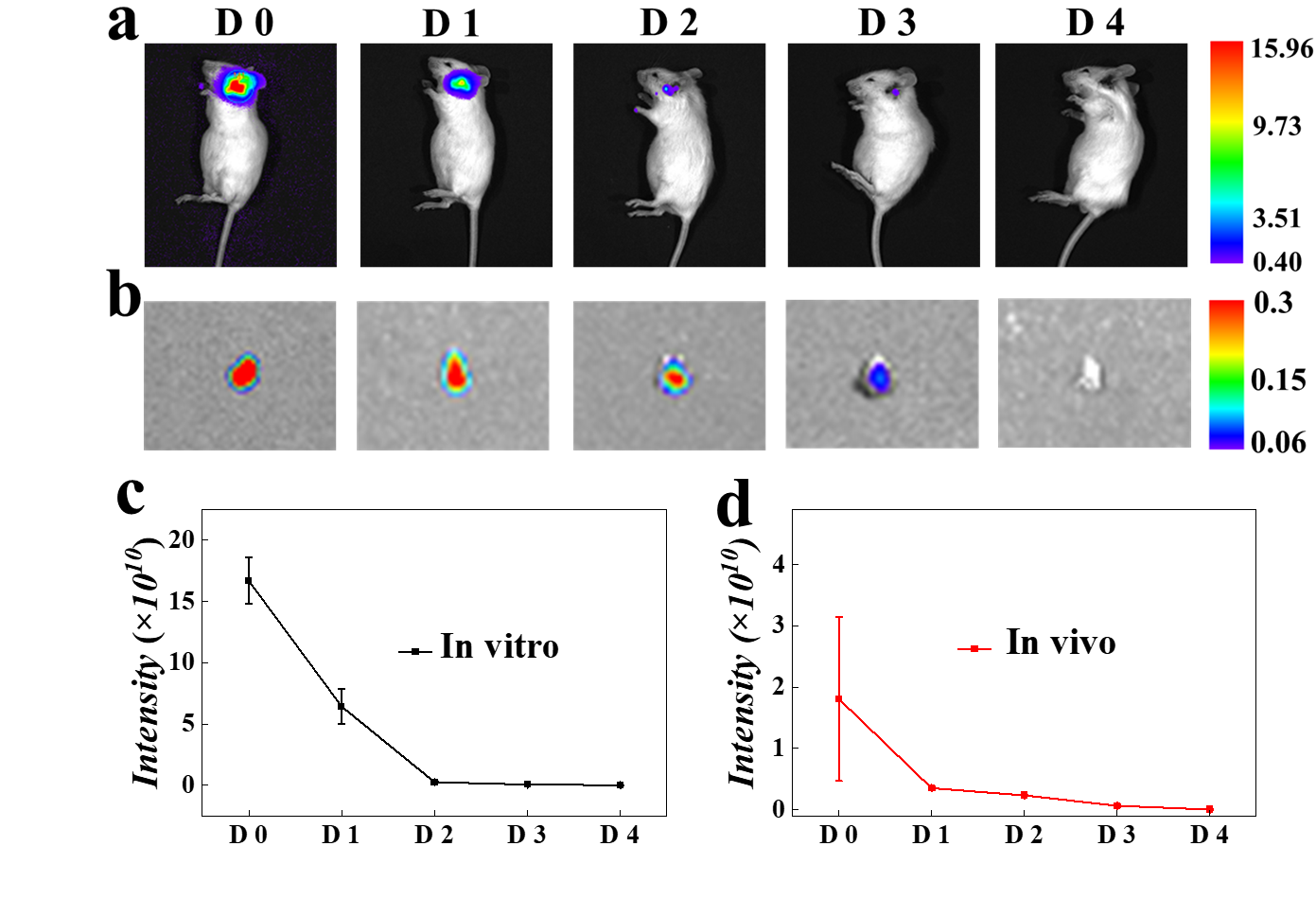


**Figure S4 (a, b)** *In vivo* and *ex vivo* cochlear IVIS pictures of the mice in the control group at different times for 4 consecutive days. **(c, d)** Statistical results of IVIS pictures.


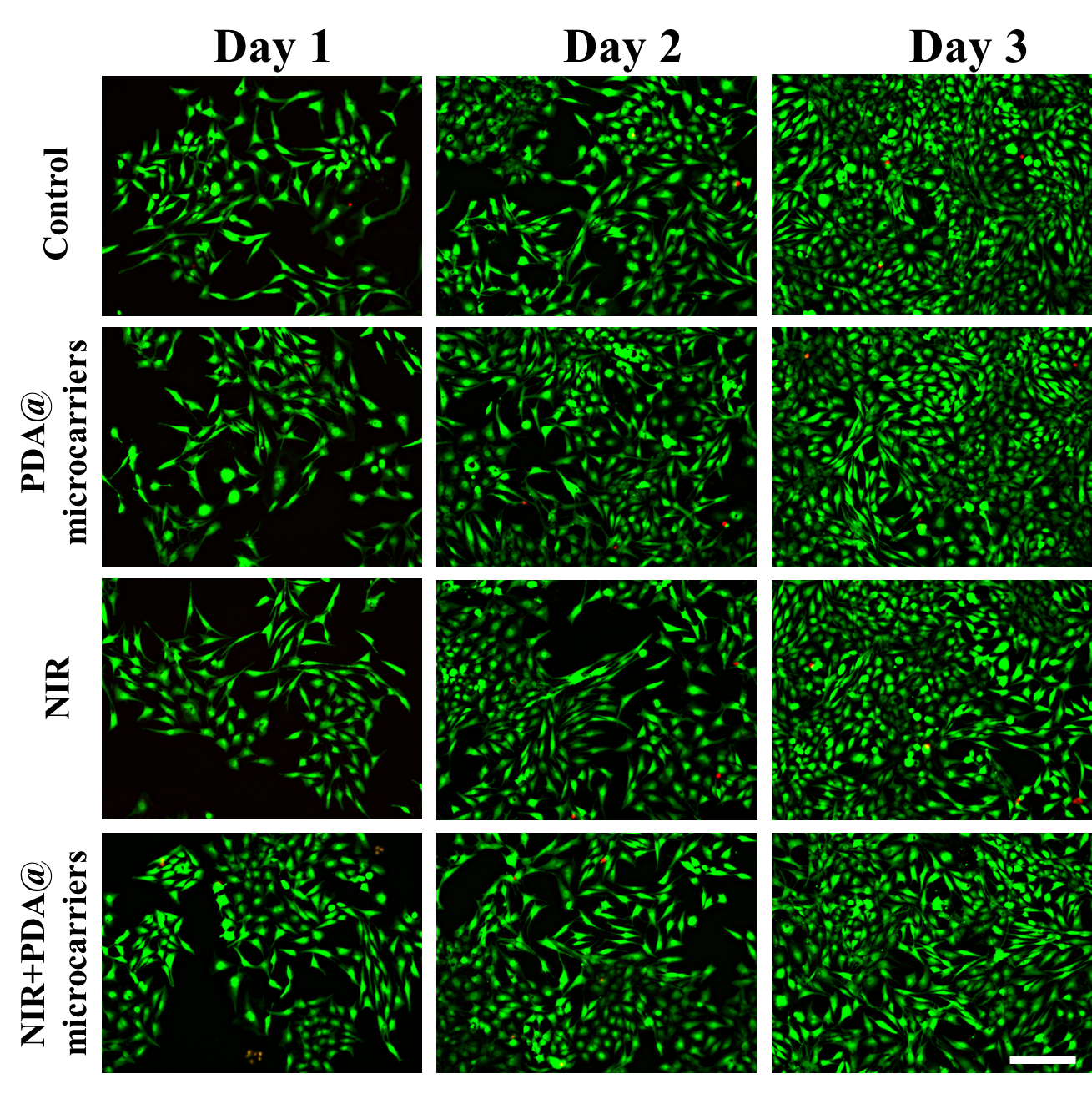


**Figure S5** Live/dead results of control, PDA@microcarriers, NIR and NIR+ PDA@microcarriers after 3 days of continuous treatment of HEI-OC1 cells. Scale bar: 250 μm.
